# The Connection between Climate Change, Surgical Care and Neglected Tropical Diseases

**DOI:** 10.5334/aogh.3766

**Published:** 2022-08-08

**Authors:** Hugh Shirley, Grace Grifferty, Elizabeth F. Yates, Nakul Raykar, Richard Wamai, Craig D. McClain

**Affiliations:** 1 Program in Medical Education, Harvard Medical School, Boston, MA, US; 2 Program in Medical Education, University of Florida College of Medicine, Gainesville, FL, US; 3 Center for Surgery and Public Health, Brigham and Women’s Hospital, Boston, MA, US; 4 Program in Global Surgery and Social Change, Harvard Medical School, Boston, MA, US; 5 Department of Cultures, Societies and Global Studies, Northeastern University, College of Social Sciences and Humanities, Integrated Initiative for Global Health, Boston, MA, US; 6 Program in Global Surgery and Social Change, Harvard Medical School, Boston, Massachusetts, US; 7 Department of Anesthesiology, Critical Care and Pain Medicine, Boston Children’s Hospital, Boston, MA, US

**Keywords:** trachoma, lymphatic filariasis, burden of disease, surgery

## Abstract

The surgical burden of neglected tropical diseases (NTDs) is set to rise alongside average temperatures and drought. NTDs with surgical indications, including trachoma and lymphatic filariasis, predominantly affect people in low- and middle-income countries where the gravest effects of climate change are likely to be felt. Vectors sensitive to temperature and rainfall will likely expand their reach to previously nonendemic regions, while drought may exacerbate NTD burden in already resource-strained settings. Current NTD mitigation strategies, including mass drug administrations, were interrupted by COVID-19, demonstrating the vulnerability of NTD progress to global events. Without NTD programming that meshes with surgical systems strengthening, climate change may outpace current strategies to reduce the burden of these diseases.

Neglected tropical diseases (NTDs) are a group of communicable and vector-borne conditions that levy a devastating human, social, and economic toll on more than 1 billion people globally. They predominantly effect marginalized populations in low- and middle-income countries (LMICs) [1,2]. Currently, the World Health Organization (WHO) prioritizes 20 NTDs, including several with surgical indications: lymphatic filariasis (LF), trachoma, cystic echinococcosis (CE), cysticercosis, snakebite envenoming, schistosomiasis, and the leishmaniases [2]. In 2019, NTDs accounted for approximately 16.5 million disability-adjusted life years (DALYs) and 104,000 deaths [3]. While pharmaceuticals are essential in both prophylaxis and treatment of many NTDs, the surgically treated sequelae of NTDs represent a significant driver of morbidity for communities in which these diseases are endemic [4]. The COVID-19 pandemic disrupted mass drug administration campaigns (MDAs) for NTD management, likely resulting in more late-stage, surgically relevant disease, demonstrating the vulnerability of these programs to disruption by global events.

Climate change, like NTDs, disproportionately impacts the world’s poor. Paradoxically, these are the communities that contribute the least to anthropogenic carbon emissions. As most NTDs are vector-borne and endemic in equatorial countries, a warming climate may expand the geographic range of some NTDs, such as LF and trachoma, while contracting the range of other NTDs to regions with environments that maintain vector-pathogen concordance [5,6]. Urbanization may drive autochthonous cases in previously disease-free regions, as the spread of some diseases is well-suited to dense habitation. Furthermore, globalization introduces pathogens to locations where suitable vectors are already present. The impact of climate change on NTDs will grow in coming years as the complex and interconnected sequelae of environmental degradation play out across the globe [5]. Climate change already affects access to key resources; clean water plays a critical role in the water, sanitation, and hygiene (WASH) strategy to eliminate several NTDs. Where clean water becomes scarcer, water usage for hygiene and disease prevention may be strained [7].

Like the disproportionate impact of climate change and NTDs on people living in LMICs, the burden of untreated surgical conditions falls most heavily on the world’s poorest and most marginalized communities [8]. In fact, morbidity and mortality from common conditions needing surgical care have grown in these regions, while the development of essential surgical care has stagnated or regressed [8]. The thematic overlaps between surgery and NTDs as neglected conditions have previously been discussed [9], but this needs updating owing to newer research. Two NTDs requiring surgical interventions, LF and trachoma, account for 2.3 million years lost to disability (YLDs) [4]. As climate change drives shifts in NTD endemicity, inadequate surgical care amplifies the burdens imposed by NTDs.

Here, we outline the interrelation and interdependence between NTDs, climate change, and surgical care, and how strategies to effectively prevent and treat NTDs must address all components. We focus here on LF and trachoma, with the inclusion of additional NTDs with surgical indications in Table 1 alongside estimates of burden of disease where data are available.

**Table 1:** NTDs with surgical complications, population living in endemic areas, any data on rate of surgical interventions for NTDs, vector, climate impact on vector, assessment of climate change risk. LF = lymphatic filariasis, CE = cystic echinococcosis, VL = visceral leishmaniasis, CL = cutaneous leishmaniasis, MCL = mucocutaneous leishmaniasis.


DISEASE	DISABILITY ADJUSTED LIFE YEARS (DALYS) GLOBALLY IN 2019		SURGICAL CASE LOAD ESTIMATES GLOBALLY PER YEAR	SURGICAL PROCEDURES	IMPACT OF CLIMATE WARMING ON PREVALENCE/TRANSMISSION

Lymphatic Filariasis	1.6 million [2]		19.43 million in 2012 [16]	Hydrocelectomy	*Anopheles* mosquitoes may have higher metabolic rate Increased geographic range of *Anopheles* with warming global temperatures

Trachoma	181 thousand [33]		2.5 million globally in 2019 [2]	Bilamellar tarsal rotation surgery (BTLS)	Impact of drought/flooding on WASH infrastructure

Cystic echinococcosis	122 thousand [33]		N/A	Liver, pancreas, etc. cystic resection	

Cysticercosis	1.37 million [33]		N/A	Ophthalmic cystic resection via various techniques, Microincision vitrectomy surgery (MIVS) Ventriculoperitoneal shunt	

Snakebite Envenoming	400,000 disabled by snakebite envenoming annually [2]		244,000–413,000 [34]	Fasciotomies, wound debridement, tissue grafts, local and free tissue flaps, and rarely amputations	Increased metabolic activity of ectotherms at higher temperatures. Increased contact between humans, snakes, and snake prey

Schistosomiasis	1.64 million [33]		N/A	Radical cystectomy, kidney transplant	Higher average temperatures may allow snails to live further from the equator.

Leishmaniases	VL	404 thousand [33]	Unknown	Splenectomy	Higher average temperatures allow sandflies to move to higher altitudes and further from the equator.

	CL/MCL	293 thousand [33]	Unknown	Cosmetic surgery to repair scarring, surgical debridement, cryosurgery	


## Lymphatic Filariasis (LF)

LF is spread by the bite of *Anopheles* and *Culex* mosquitoes infected with *Wuchereria bancrofti* or *Brugia* [10]. LF is endemic to parts of Sub-Saharan Africa, South America, and Southeast Asia, exposing an estimated 857 million people to infection [10]. The most common clinical presentation of chronic *W. bancrofti* infection in males is a hydrocele, a stigmatizing condition that forms when *W. bancrofti* nematodes damage and block lymphatic vessels in the scrotum. People with hydroceles on average generate less income and engage in less social activity than community members without hydroceles [11]. Hydroceles require surgical intervention for definitive treatment [10,12]. Up to 83% of the total economic cost of LF in India and Africa were estimated to be attributable to hydrocele, totaling approximately US$2 billion [13]. A cost-benefit analysis of LF hydrocelectomies in Malawi found a ratio of 24.8, suggesting surgeries were more cost-effective than HIV therapy and as effective as hernia repair [14].

In 2019, 547,953 hydrocele cases across 56 of 72 endemic countries were reported to the WHO Programme to End LF (GPELF) [15]. This number is likely an underestimate due to the stigmatizing nature of the condition and challenges with data gathering in remote areas, demonstrated by the lack of reported cases in 16 endemic countries. Indeed, there were an estimated 19 million cases of hydrocele in 2020, suggesting an enormous discrepancy between the expected and reported number of cases that cannot be explained by current surgical capacity or mass drug administration (MDA) campaigns alone [16,17]. Most people with hydroceles require hydrocelectomy for treatment, and continued efforts to treat these patients are essential for both reducing disease burden and for surveillance.

While MDAs have successfully reduced the global burden of LF since establishment in 2000, interruption of the parasite’s transmission cycle has not yet been achieved in many countries. Medical treatment must be administered early in the disease process to prevent hydrocele formation, as scrotal swelling will not resolve with medical therapy alone after development. Up to 90% of men over 70 years old living along the endemic east coast of Kenya and Tanzania have hydroceles, suggesting a high prevalence of LF and high likelihood of developing hydrocele with longstanding exposure [18]. The morbidity associated with this condition will likely increase as demographics shift towards older and more chronically exposed populations [19].

As endemic regions experience warming of average temperatures, *Anopheles* mosquitoes are likely to increase their geographic reach further from the equator, introducing disease to previously unaffected populations [20]. One ecological model combining demographic and climatic variables projected the increase of people at risk of LF infection in Africa alone from 543–804 million to an astounding 1.65–1.86 billion by 2050 [21]. With dissemination of the LF vector, the burden of LF hydrocele should be expected to rise globally unless programs to prevent LF and treat its complications are scaled up accordingly.

## Trachoma

Trachoma is an eye infection caused by *Chlamydia trachomatis*, an obligate intracellular bacterium spread by *Musca sorbens* flies or by hand-eye contact. Active trachoma involves an inflammatory conjunctivitis that can lead to trachomatous trichiasis (TT)—an in-turning of the upper eyelid resulting in cornea trauma from eyelash contact that, untreated, leads to blindness [22]. Globally, trachoma is responsible for blindness or visual impairment in about 1.9 million people and 181,000 DALYs, making it the leading cause of preventable blindness worldwide [23]. TT affected approximately 2.5 million people in 2019, with over 1.5 million of those in Africa [2]. Trachoma and TT impose a high economic burden on individuals and communities, with lost productivity of US$8 billion [23].

Surgery is the first step in trachoma elimination programs focusing on a SAFE (surgery, antibiotics, facial cleanliness, and environmental improvement) strategy first introduced in 1996 to reduce disease burden [24]. Through successful intervention programs, the number of people at risk for trachoma has decreased by 91% and the number of people requiring surgery has decreased by 68% [25]. Nonetheless, the WHO acknowledges that critical action is needed to eliminate trachoma, which depends on access to high-quality surgical care and outcomes management post-surgery [2]. Surgery is essential to prevent blindness, and can be accomplished using either of two straightforward procedures that can be cost-effectively implemented in field settings with local anesthesia and minimal recovery time: posterior lamellar (PLTR) or bilamellar tarsal rotation (BLTR) [26].

The facial cleanliness and environmental improvement components of SAFE are both impacted by climate change. Improving access to WASH promotes facial cleanliness but requires resilient infrastructure. Prolonged and more frequent periods of drought, flooding, and other factors that are expected to increase with climate change will stress community water supplies and ultimately inhibit trachoma control. Communities burdened by trachoma are often in arid and water-limited settings, necessitating investment in sustainable water infrastructure that prioritizes equity and safety [27]. Environmental improvement refers to sanitation and waste management; activities that reduce fly burden and limit the spread of trachoma. These interventions become critical as the fly vector for trachoma thrives in hot, dry climates [28]. Poorer trachoma control will lead to increased numbers of TT cases, further increasing the need for surgery.

## Conclusion

Climate change is likely to have variable impacts on each NTD depending on vector and pathogen biology and human activity [5,29]. While rising average temperatures are a significant consequence of anthropogenic climate change, additional human-driven changes, such as conflict and migration, will play a role in the future distribution and impact of NTDs. Conflict, potentially over resource scarcity in a warmer world, may disrupt MDA campaigns, damage healthcare infrastructure, and drive migration, additionally amplifying infection risk within unhygienic, crowded refugee camps [29]. Applying methods to predict surgical demands due to impacts of conflict and climate change is necessary to forecast needs [30]. Climate-driven sociopolitical destabilization and lack of investment in resilient healthcare infrastructure will challenge current gains in the fight against NTDs and threaten achievement of the 2030 goals [31].

Interruption of MDAs, for even a year, as has happened due to COVID-19, could contribute to rebounds in several NTDs and increase surgical demand [32]. The success of decades of surveillance and preventative pharmacotherapy initiatives are vulnerable to global events, meaning surgical systems strengthening is critical for a resilient NTD response. The dual benefit of planning for the surgical sequalae of NTDs is the fortification of communities against any lapse in MDA programming for NTDs while simultaneously equipping communities with tools and resources for treating primary surgical conditions, such as those requiring bellwether procedures [8]. Surgical cases of NTDs must be incorporated into surveillance and intervention planning, as they continue to drive morbidity and mortality in endemic regions.

Additional research is needed to better establish risk factors for progression of NTDs into surgical cases. Temporospatial analysis of surgical burden of NTDs requires prospective surveillance to incorporate climate variables and to establish reliable reporting systems. Incorporation of surgical services into MDAs and other campaigns should be considered for relevant NTDs, as well as into national health policy priorities. Research into the entomologic vectors of each NTD, as well as the interactions between vector and pathogen, could lead to better models for predicting the evolving boundaries of each disease. Most critical, though, is the expansion of surgical care to mitigate the current underappreciated burden of surgical NTDs and to prepare communities for any increase in surgical sequelae of NTDs brought on by anthropogenic climate change.
